# Efficacy and Safety of Traditional Chinese Medicine Exercise Versus Oral Medication in the Treatment of Neck Pain: Study Protocol for a Systematic Review and Meta-Analysis

**DOI:** 10.2196/86168

**Published:** 2026-05-18

**Authors:** Guancheng Wang, Yuanming Zhong, Shichang Yang, Haoran Sun, Jie Si, Yan Long, Jinyu Li

**Affiliations:** 1 Department 1, Guangxi University of Chinese Medicine, Nanning City, China; 2 Orthopedics Department, The First Affiliated Hospital of Guangxi University of Traditional Chinese Medicine, 89-9 Dongge Road, Qingxiu District, Nanning City, Guangxi Zhuang Autonomous Region, China, Nanning City, 530023, China, 86 13507715643

**Keywords:** traditional Chinese medicine exercises, neck pain, cervical spondylosis, Baduanjin, Tai Chi, systematic review, meta-analysis, drug therapy

## Abstract

**Background:**

Neck pain poses a significant and growing public health challenge, with rising prevalence among younger populations and negative impacts on both quality of life and socioeconomic costs. Clinical manifestations are diverse, including restricted movement, muscle spasms, headaches, and upper limb numbness. Although drug therapy is widely used, its long-term use is limited by adverse effects. Traditional Chinese medicine (TCM) exercises offer a promising alternative, but high-quality evidence directly comparing their efficacy and safety to oral medications is currently lacking.

**Objective:**

This study aims to compare the efficacy and safety of TCM exercises and oral medication in treating neck pain.

**Methods:**

We will identify relevant randomized controlled trials (RCTs) through a systematic search of multiple databases (including PubMed, Embase, Cochrane Library, Web of Science, China National Knowledge Infrastructure [CNKI], Chinese Biomedical Literature Database [CBM], VIP, and Wanfang) from inception through September 2025. Only RCTs directly comparing TCM exercise to oral medication will be included. Study quality will be assessed using the Cochrane RoB2 tool, and the overall evidence will be graded via the Grading of Recommendations Assessment, Development and Evaluation (GRADE) approach. For heterogeneity, the *I*² statistic and Cochran Q test will be applied. A fixed-effect model will be adopted if *I*²<50% and *P*≥.10; otherwise, subgroup analysis will be performed. Should heterogeneity persist, sensitivity analysis or a random-effects model will be employed, leading to a reduction in the GRADE rating.

**Results:**

This is a study protocol; therefore, no results are available at this stage. The systematic review is scheduled to commence in August 2025, with the literature search from August to September 2025, study screening from September to December 2025, data extraction and analysis from January to May 2026, and manuscript submission by June 2026.

**Conclusions:**

This protocol outlines a systematic review and meta-analysis designed to compare the efficacy and safety of TCM exercises versus oral medications for neck pain. The completed review aims to synthesize the available evidence and clarify whether TCM exercises offer a comparable or superior alternative to pharmacotherapy. By systematically evaluating direct head-to-head RCTs, this study seeks to provide evidence-based insights to inform clinical decision-making. Potential limitations of the forthcoming review may include heterogeneity in exercise protocols, challenges in blinding, and a possible limited number or geographic concentration of available trials, which could affect the generalizability of the findings. These limitations will be considered when interpreting the results.

## Introduction

Neck pain is one of the four common diseases worldwide and has become a significant public health issue that threatens personal health and reduces the quality of life [1,2]. The symptoms of neck pain are diverse, including restricted movement of the cervical spine and muscle spasms, which may also cause headaches, numbness in the upper limbs, and in severe cases, even affect sleep quality and work ability [3,4]. The causes of the disease are complex and are mostly related to muscle strain, soft tissue injuries, cervical joint disorders, and degeneration of cervical intervertebral discs [5,6]. At present, there is still no standardized treatment plan for this disease, and clinical practice mainly relies on drug therapy [7,8]. In this context, the course of neck pain often shows a high degree of protracted nature. Approximately half of the patients’ acute pain will turn into chronic pain, which makes it urgent for clinicians to find treatment options that can effectively control neck pain for a long term [9,10].

As a commonly used treatment method in clinical practice, drug treatment for neck pain mainly exerts its therapeutic effect in pain relief. However, it has limited effects on improving cervical spine function and restoring cervical spine curvature [11,12]. Furthermore, the drug treatment for neck pain has significant limitations. It often comes with adverse reactions such as gastrointestinal bleeding, drowsiness addiction, etc, which restricts its long-term application in clinical practice [13,14]. This has made it an urgent need in clinical practice to actively explore nonpharmacological intervention methods, further highlighting the clinical value of nonpharmacological intervention approaches such as traditional Chinese medicine (TCM) exercises [15,16]. Due to its low-risk, noninvasive and proven-effective characteristics, TCM exercises are increasingly playing a significant role in the prevention and rehabilitation management of neck pain [17-19]. TCM exercises refer to a type of active rehabilitation and preventive measure that involves the coordinated efforts of body movements, breathing and exhalation, as well as mental regulation. Through these activities, it aims to unblock meridians, regulate blood and qi, coordinate internal organs, strengthen muscles and bones, and calm the mind and soothe the spirit [17]. Common TCM exercises include Baduanjin, Yijinjing, Tai Chi and Wuqinxi. Their excellent versatility can meet the health and fitness needs of different audiences [10,15,20]. Interestingly, the benefits brought by TCM exercises extend beyond the physical level. Through regular practice, one can achieve both physical and mental improvement, alleviating anxiety while also reducing anxiety’s physical manifestations [21,22]. This characteristic enables practitioners to achieve a significant sense of participation accomplishment, which not only ensures good compliance but also constitutes its core advantage in the field of public health promotion [23,24].

Although both TCM exercises and drug therapy have been used in clinical practice, there is still a lack of systematic comparison in terms of efficacy and safety between the two. Furthermore, there are still several key issues that require in-depth exploration, such as the differences in therapeutic effects among various TCM exercise routines, and the impact of standardized intervention plans on the therapeutic outcomes. This protocol outlines a systematic review that will comprehensively compare the efficacy and safety of TCM-based exercise and medication in treating neck pain, aiming to provide evidence-based guidance for the selection of clinical treatment plans.

## Methods

### Study Registration

This systematic review protocol was formulated in accordance with Preferred Reporting Items for Systematic Review and Meta-Analysis Protocols (PRISMA-P) and has been registered in the PROSPERO database (CRD 420251156106).This protocol elaborates on the planned research criteria, database selection, data collection, and bias assessment methods, aiming to ensure the objectivity, fairness, transparency, and repeatability of the forthcoming systematic review. If there are any changes in the research methods, the protocol will be updated in the PROSPERO database.

### Ethics and Dissemination

As the data for this study will be sourced exclusively from published literature and will not involve direct data collection from patients, ethical approval is not required for this systematic review. The findings of the completed review are anticipated to clarify the comparative efficacy and safety of TCM exercise therapy versus medication for neck pain, thereby offering evidence-based guidance to inform clinical decision-making.

### Search Strategy

We will systematically search the following databases: China National Knowledge Infrastructure (CNKI), VIP, Web of Science, PubMed, Cochrane Library, Embase, Chinese Biomedical Literature Database (CBM), and Wanfang Data Knowledge Service Platform. The search period will cover results from the establishment of each database to September 2025, aiming to collect relevant randomized controlled trials (RCTs). The search strategy follows the framework of “P (research subject) + I (intervention measure) + S (research design),” and will combine the use of Medical Subject Headings (MeSH) and free words for construction. Through the logical operators “OR” and “AND,” the search scope will be expanded and the accuracy will be improved. To include the latest studies, the team will conduct another search of the above databases one month before submission, and update the final search date to this point. The specific search strategy of PubMed is shown in Table 1.

**Table 1.** Inclusion and exclusion criteria.

Category	Inclusion criteria	Exclusion criteria
Population	Patients aged 18‐70 years with neck pain (including chronic nonspecific neck pain, cervical disc herniation, and cervical degenerative diseases) VAS^a^ score >3 NDI^b^ score >10 No history of shoulder or neck surgery	Patients aged <18 or >70 years Severe systemic diseases (eg, cardiovascular, cerebrovascular, malignant tumors, liver/kidney dysfunction) Spinal cord type cervical spondylosis or nerve root structural damage Pregnant or lactating women Previous shoulder/neck surgery Communication disorders or the inability to cooperate
Intervention	TCM^c^ exercises defined as mind-body practices rooted in TCM theory: Baduanjin (Eight-Section Brocade) Tai Chi (Taijiquan) Yijinjing (Muscle-Tendon Change Classic) Wuqinxi (Five-Animal Play)	Modern physiotherapeutic or general neck exercises Exercises not explicitly derived from or named as recognized TCM exercise forms
Comparison	Oral drug treatment only: NSAIDs^d^ Muscle relaxants Other analgesics	No-treatment control Wait-list control Placebo control Any other nonpharmacological comparator
Outcomes	Primary outcome: change in VAS score Secondary outcome: NDI score SAS^e^ score Cobb angle Adverse events	Studies not reporting any of the specified outcomes
Study design	Randomized controlled trials (RCTs) with direct head-to-head comparison between TCM exercise and oral medication. For 2-arm trials that include a TCM exercise arm, a drug arm, and a placebo or blank control arm, only the direct comparison between the TCM exercise and drug arms will be extracted for analysis. No language or publication status restrictions No sample size restrictions	Non-RCTs (cohort studies, case-control studies, cross-sectional studies) Reviews and meta-analyses Case reports or case series Observational studies Studies with only a blank/placebo control and no direct drug comparator Conference abstracts without full text

a VAS: visual analogue scale.

b NDI: Neck Disability Index.

c TCM: traditional Chinese medicine.

d NSAID: nonsteroidal anti-inflammatory drug.

e SAS: Self-Rating Anxiety Scale.

### Study Selection Process and Data Extraction

#### Study Selection

This study will adhere to the principle of independent double screening by two individuals and arbitration by a third party to ensure the objectivity and accuracy of the screening process. Two researchers (HS and YL) who have received systematic training will independently conduct the screening of titles, abstracts, and full texts. If there is a disagreement, it will first be resolved through negotiation; if negotiation fails, it will be referred to a third-party expert (YZ) for arbitration. The arbitration result and reasons must be recorded and archived. For complex disputes such as incomplete information or boundary cases of standards, a three-person group meeting will make a collective decision. The entire process will be tracked and recorded using the NoteExpress software, and a flowchart will be drawn based on the Preferred Reporting Items for Systematic reviews and Meta-Analyses (PRISMA) guidelines to clearly display the literature screening situation at each stage.

#### Data Extraction

To systematically evaluate the efficacy and safety of the treatment and control for potential methodological heterogeneity, we have designed a standardized data extraction process. Two researchers will independently extract the data from the included literature, covering key variables that might affect the research results. For the TCM exercise group, the extraction items will include exercise frequency (times/day or times/week), single duration (minutes), treatment course, and guidance method (online or offline). For the drug therapy group, the extraction items will include drug formulation and specification, frequency of administration (times/day), and total treatment duration (days/weeks). All variables will be standardized coded in the form of categorical, continuous or ordinal data. In case of disagreement during the extraction process, it will first be resolved through consultation between two researchers; if the consultation fails, it will be submitted to a senior researcher (YZ) with ten years or more of traditional medicine research experience for arbitration. The arbitration must be based on domain consensus and research design principles, and the reasons must be recorded; if there is still a dispute, it will be resolved through a team special meeting, and the meeting minutes will be archived to ensure the standardization and traceability of the extraction process.

### Risk of Bias Assessment

Two evaluators (GW and HS) will independently use the Cochrane RoB 2.0 tool to assess the risk of bias for the included RCTs. The assessment will cover five domains: bias arising from the randomization process, bias due to deviations from intended interventions, bias due to missing outcome data, bias in measurement of the outcome, and bias in selection of the reported result. Each domain will be classified as “low risk,” “some concerns,” or “high risk” of bias, leading to an overall risk of bias judgment for each study. The overall quality of the evidence will be graded using the Grading of Recommendations Assessment, Development and Evaluation (GRADE) system. The evidence grade will be downgraded under the following circumstances: more than half of the studies have a high risk of bias; after subgroup analysis, the heterogeneity remains high (*I*²≥50% and Cochran Q test *P*<.10) and cannot be reasonably explained; the study population, intervention, or outcome is significantly indirect to the target issue; or there is publication bias (such as asymmetry in the funnel plot, and negative results are not reported). Under certain conditions, the evidence level can be upgraded. For instance, when there is a large effect size, a clear dose-response relationship (such as the frequency of Qigong exercises being related to the degree of pain relief), or all biases point to an underestimation of the true effect. Any disagreements that arise during the evaluation process will first be resolved through negotiation among the evaluators. If no consensus is reached, a third party (HS) will arbitrate. A pre-evaluation will be conducted before the formal assessment to unify the standards. The final bias risk assessment results will be presented in a chart format. All quality evaluation data of the included studies will be used for subsequent meta-analysis or bias risk analysis.

### Statistical Analysis

We will conduct a meta-analysis using RevMan 5.4 software. The *I*² statistic and Cochran Q test will be used to assess heterogeneity among studies. If *I*²<50% and *P*≥.10, indicating low heterogeneity, a fixed-effect model will be applied. If *I*²≥50% or *P*<.10, indicating substantial heterogeneity, we will first explore potential sources through subgroup analysis before considering a random-effects model. To investigate potential sources of heterogeneity, we will conduct exploratory subgroup analyses if sufficient data are available (defined as at least two studies per subgroup), including analyses by drug type (nonsteroidal anti-inflammatory drugs vs muscle relaxants vs other analgesics), intervention duration (≤12 weeks vs >12 weeks), TCM exercise type (Baduanjin vs Tai Chi vs other forms), baseline pain severity (moderate visual analogue scale [VAS] score ≤5.4 vs severe VAS score >5.4, based on established cut-offs for chronic pain), geographic region (Asia vs non-Asia), and study quality (low risk of bias vs high risk/some concerns as assessed by RoB 2). These analyses will be interpreted with caution, particularly if the number of included studies is limited.

We acknowledge that exercise “intensity” is complex and often poorly reported in nonpharmacological trials. While total weekly exercise time (minutes/week) will be extracted, its use as a sole proxy for intensity has significant limitations. Therefore, a formal subgroup analysis based on predefined intensity cut-offs will not be performed. Instead, if considerable heterogeneity remains, we will explore the distribution of weekly exercise time across studies descriptively.

If heterogeneity cannot be adequately explained through subgroup analyses, a random-effects model will be used for pooling, and the evidence level will be downgraded accordingly using the GRADE approach. Statistical significance will be set at *P*<.05 for overall effect estimates.

### Multiple Outcomes

This review includes one primary outcome and several secondary outcomes, raising the potential issue of multiplicity and inflated type I error. To address this, our interpretation will follow a hierarchical approach: the primary conclusion will be drawn from the primary outcome (change in VAS score), while secondary outcomes will be considered supportive and hypothesis-generating.

We will not apply formal statistical corrections such as Bonferroni, as these methods are overly conservative for correlated outcomes in systematic reviews and may obscure clinically important findings. Instead, all outcomes will be presented with 95% confidence intervals, and the certainty of evidence for each outcome will be assessed using the GRADE framework. This approach provides a more nuanced evaluation than simple *P* value thresholds. As a sensitivity analysis, we will consider the false discovery rate approach to explore the potential impact of multiple testing, but final conclusions will be based on the overall body of evidence, including effect size, consistency, and GRADE assessment.

### Publication Bias

Based on the number of included RCTs, the corresponding evaluation method will be selected.

If the number of included RCTs is less than 10, we will use the RevMan software to draw a funnel plot. By observing the symmetry of the graph, the publication bias can be determined: a symmetrical funnel plot indicates a low risk of publication bias; an asymmetrical one suggests that publication bias may exist. It will be necessary to analyze the reasons in combination with clinical common sense (eg, negative result studies have not been published).

If the number of included RCTs is ≥10, based on the funnel plot, Stata software will be used to conduct an Egger linear regression test and Begg test. The criterion for determining significant publication bias is *P*<.05. If there is publication bias, the “trim-and-fill method” will be used to estimate the impact of potential missing studies on the combined effect size and evaluate the robustness of the conclusion.

## Results

This is a study protocol for a systematic review and meta-analysis; therefore, no results are available at the time of publication. The systematic review is scheduled to commence in August 2025. The anticipated timeline is as follows: literature search from August to September 2025; study screening from September to December 2025; data extraction and quality assessment from January to March 2026; statistical analysis and evidence synthesis from April to May 2026; and manuscript submission by June 2026. Any deviations from this timeline will be reported in the final publication.

## Discussion

Neck pain is the fourth leading cause of disability worldwide, with an annual prevalence rate exceeding 30%, and represents a major contributor to labor loss and disability [25]. Its incidence is increasingly observed in younger populations, imposing substantial economic burdens on families and society [3,26]. Currently, no unified or standardized treatment protocol for neck pain exists [27]. While pharmacological therapy remains the conventional approach, nonpharmacological interventions—particularly TCM exercises—have attracted growing clinical interest due to their noninvasive nature and potential for long-term management [28,29]. However, whether TCM exercises are comparable to or more effective than oral medications in terms of both efficacy and safety remains to be systematically evaluated.

However, it should be noted that although TCM exercise has a wide variety of forms, its efficacy largely depends on individual differences [10,30]. During the clinical application process, the duration, frequency, and intensity of traditional Chinese physical therapy exercises are mostly determined by the physicians, and there are various versions of traditional Chinese physical exercises, lacking a unified standard [30,31]. This “empirical” approach to scheme formulation has led to significant differences in the application effects of the same therapy in different medical institutions and under the hands of different physicians, making it difficult to establish a replicable treatment model [32]. In addition, the differences in the effectiveness of TCM exercises between the East and the West are rooted in cultural acceptance [33,34]. In Asia, it is highly compatible with the cultural DNA of the region; however, in Europe and America, most people find it difficult to understand TCM culture, which consequently weakens the therapeutic effect [35].From a long-term management perspective, TCM exercises are generally considered to have a favorable safety profile compared to pharmacological interventions [36]. The forthcoming review will systematically assess whether the available evidence supports this assumption and whether TCM exercises can be recommended as a viable long-term strategy for chronic nonspecific neck pain. If confirmed, such findings could have implications for shifting the clinical focus from symptomatic treatment toward sustained prevention and rehabilitation. To address the cultural heterogeneity observed and enhance the global applicability of TCM exercises, future research and clinical implementation should consider strategies for cultural adaptation. This goes beyond mere translation of instructions. Potential approaches include: (1) contextualizing the practice within local wellness or mindfulness frameworks that are more familiar to non-Asian populations; (2) modifying exercise names, metaphors, and imagery to align with culturally resonant concepts (eg, emphasizing “mindful movement,” “stress reduction,” or “gentle stretching” rather than solely “meridian regulation”); (3) involving community stakeholders and target cultural groups in the design and refinement of intervention protocols to ensure relevance and acceptability; and (4) training instructors who are not only proficient in the exercises but also culturally competent in delivering them to diverse populations. By proactively integrating such cultural adaptation strategies, the external validity and effectiveness of TCM exercises in multinational trials and real-world settings outside Asia could be significantly improved.

As mentioned, this systematic review has not yet been performed. However, based on our preliminary understanding of the existing literature, we anticipate potential challenges in the forthcoming review. Methodological limitations in the primary studies are likely, such as small sample sizes, unclear reporting of randomization and allocation concealment, and challenges in blinding participants and personnel due to the nature of exercise interventions, all of which could affect the reliability of individual study results. Significant clinical and methodological heterogeneity is also expected due to variations in TCM exercise protocols (eg, type, frequency, duration), drug regimens, and outcome assessment tools, which may complicate data synthesis and interpretation. Furthermore, we are aware that the current body of evidence may be limited in volume; based on scoping searches, it is possible that most eligible trials have been conducted in Asian populations. If this geographic concentration emerges in the final included studies, coupled with potential differences in cultural acceptance of TCM practices and local health care contexts, it may limit the generalizability of our findings to non-Asian settings. Our planned subgroup analysis by geographic region (Asia vs non-Asia) will formally explore this aspect if sufficient data are available.

Given the limitations of this study, future primary studies should consider more high-quality, large-sample, multicenter head-to-head RCTs designed to clarify the differences in efficacy and safety between TCM exercise and drug treatment in different disease courses and various types of neck pain. Second, a unified intervention plan for TCM exercise (such as standard movements, practice frequency and duration in TCM) and evaluation criteria for efficacy should be formulated to reduce heterogeneity among studies. Third, long-term follow-up studies (≥1 year) should be conducted to assess the long-term efficacy, recurrence rate, and safety of TCM exercise and drug treatment. Finally, the effects of combined treatment with TCM exercise and drugs should be explored, and the applicable population and optimal treatment plan for combined treatment should be clarified to provide more comprehensive evidence-based basis for clinical treatment.

## Supplementary material

10.2196/86168 Multimedia Appendix 1 Visual Abstract

## Abbreviations

CBM: Chinese Biomedical Literature Database
CNKI: China National Knowledge Infrastructure
GRADE: Grading of Recommendations Assessment, Development and Evaluation
MeSH: Medical Subject Headings
PRISMA: Preferred Reporting Items for Systematic reviews and Meta-Analyses
PRISMA-P: Preferred Reporting Items for Systematic Review and Meta-Analysis Protocols
RCT: randomized controlled trial
TCM: traditional Chinese medicine
VAS: visual analogue scale

## References

[1] Li G, Wang C, Tian C (2025). Trends in global musculoskeletal rehabilitation needs from 1990 to 2050: a systematic analysis of the Global Burden of Disease Study 2021. Arch Phys Med Rehabil.

[2] Safiri S, Kolahi AA, Hoy D (2020). Global, regional, and national burden of neck pain in the general population, 1990-2017: systematic analysis of the Global Burden of Disease Study 2017. BMJ.

[3] Kazeminasab S, Nejadghaderi SA, Amiri P (2022). Neck pain: global epidemiology, trends and risk factors. BMC Musculoskelet Disord.

[4] Mingels S, Granitzer M, Jull G, Dankaerts W (2025). The occurrence of cervicogenic headache: a mapping review. Musculoskelet Sci Pract.

[5] Kim R, Wiest C, Clark K, Cook C, Horn M (2018). Identifying risk factors for first-episode neck pain: A systematic review. Musculoskelet Sci Pract.

[6] Binder AI (2007). Cervical spondylosis and neck pain. BMJ.

[7] Hogg-Johnson S, van der Velde G, Carroll LJ (2008). The burden and determinants of neck pain in the general population: results of the Bone and Joint Decade 2000-2010 Task Force on Neck Pain and Its Associated Disorders. Spine (Phila Pa 1976).

[8] Kjaer P, Kongsted A, Hartvigsen J (2017). National clinical guidelines for non-surgical treatment of patients with recent onset neck pain or cervical radiculopathy. Eur Spine J.

[9] de Zoete RM, Armfield NR, McAuley JH, Chen K, Sterling M (2020). Comparative effectiveness of physical exercise interventions for chronic non-specific neck pain: a systematic review with network meta-analysis of 40 randomised controlled trials. Br J Sports Med.

[10] Gao Q, Li X, Pan M (2024). Comparative efficacy of mind-body exercise for treating chronic non-specific neck pain: a systematic review and network meta-analysis. Curr Pain Headache Rep.

[11] Peloso P, Gross A, Haines T (2007). Medicinal and injection therapies for mechanical neck disorders. Cochrane Database Syst Rev.

[12] Huo L, Liu G, Deng B (2024). Effect of use of NSAIDs or steroids during the acute phase of pain on the incidence of chronic pain: a systematic review and meta-analysis of randomised trials. Inflammopharmacology.

[13] Wongrakpanich S, Wongrakpanich A, Melhado K, Rangaswami J (2018). A comprehensive review of non-steroidal anti-inflammatory drug use in the elderly. Aging Dis.

[14] Huang JF, Meng Z, Zheng XQ (2020). Real-world evidence in prescription medication use among U.S. adults with neck pain. Pain Ther.

[15] Lauche R, Wayne PM, Fehr J, Stumpe C, Dobos G, Cramer H (2017). Does postural awareness contribute to exercise-induced improvements in neck pain intensity? A secondary analysis of a randomized controlled trial evaluating Tai Chi and neck exercises. Spine (Phila Pa 1976).

[16] Castellini G, Pillastrini P, Vanti C (2022). Some conservative interventions are more effective than others for people with chronic non-specific neck pain: a systematic review and network meta-analysis. J Physiother.

[17] Liu X, Pan F, Wang Q, Wang S, Zhang J (2024). Traditional Chinese Rehabilitation Exercise (TCRE) for myofascial pain: current evidence and further challenges. J Pain Res.

[18] Kong L, Ren J, Fang S, He T, Zhou X, Fang M (2022). Traditional Chinese exercises on pain and disability in middle-aged and elderly patients with neck pain: a systematic review and meta-analysis of randomized controlled trials. Front Aging Neurosci.

[19] Lauche R, Stumpe C, Fehr J (2016). The effects of Tai Chi and neck exercises in the treatment of chronic nonspecific neck pain: a randomized controlled trial. J Pain.

[20] Shao Y, Han JY, Li HL, Ren ZP, Yang H (2024). Effect of traditional Chinese exercises on the physical and mental health of stroke patients: a meta-analysis. Front Neurol.

[21] Lu Y, Li J, Ni W (2023). Effectiveness of mind-body exercise via Baduanjin on physical and psychological outcomes in patients with pulmonary ground-glass nodules: a non-randomized controlled pilot study. Complement Ther Clin Pract.

[22] Wang X, Luo H (2024). Effects of traditional Chinese exercise therapy on pain scores, sleep quality, and anxiety-depression symptoms in fibromyalgia patients: a systematic review and meta-analysis. BMC Musculoskelet Disord.

[23] Yeung A, Lepoutre V, Wayne P (2012). Tai chi treatment for depression in Chinese Americans: a pilot study. Am J Phys Med Rehabil.

[24] Yu Y, Wu T, Wu M (2024). Evidence map of traditional Chinese exercises. Front Public Health.

[25] Cohen SP (2015). Epidemiology, diagnosis, and treatment of neck pain. Mayo Clin Proc.

[26] Fu F, Liu B, Gu H (2025). Temporal trends in neck pain prevalence among adolescents and young adults aged 10-24 from 1990 to 2019. Arch Med Sci.

[27] Xia W, Liu J, Liu C (2024). Burden of neck pain in general population of China, 1990-2019: an analysis for the Global Burden of Disease Study 2019. J Glob Health.

[28] Hoy D, March L, Woolf A (2014). The global burden of neck pain: estimates from the global burden of disease 2010 study. Ann Rheum Dis.

[29] Corp N, Mansell G, Stynes S (2021). Evidence-based treatment recommendations for neck and low back pain across Europe: a systematic review of guidelines. Eur J Pain.

[30] Dong Y, Kuang X, Dong L (2023). Exploring the efficacy of traditional Chinese medicine exercise in alleviating anxiety and depression in older adults: a comprehensive study with randomized controlled trial and network meta-analysis. Front Psychol.

[31] de Oliveira-Souza AIS, Barbosa-Silva J, Gross DP (2025). Comparative effectiveness of manual therapy, pharmacological treatment, exercise therapy, and education for neck pain (COMPETE study): protocol of a systematic review with network meta-analysis. Syst Rev.

[32] Dai M, Luo Z, Hu S (2023). Effects of traditional Chinese exercises on the rehabilitation of patients with chronic heart failure: a meta-analysis. Front Public Health.

[33] Tang H, Huang W, Ma J, Liu L (2018). SWOT analysis and revelation in traditional Chinese medicine internationalization. Chin Med.

[34] Chen T, Wang Y, Xie R, Dong L, Chen J, Yang L (2025). Global research on the treatment of cancer patients during the COVID-19 pandemic: visualisation and bibliometric analysis. Clin Oncol (R Coll Radiol).

[35] Yang LH, Phelan JC, Link BG (2008). Stigma and beliefs of efficacy towards traditional Chinese medicine and Western psychiatric treatment among Chinese-Americans. Cultur Divers Ethnic Minor Psychol.

[36] Tian Y, Liu ZY, Wang JH, Qian JH (2025). The efficacy and safety of Baduanjin exercise as complementary therapy for pain reduction and functional improvement in knee osteoarthritis: a meta-analysis of randomized controlled trials. Complement Ther Med.

