# Supplementary figures and images for: Efficacy and Safety of Traditional Chinese Medicine Exercise Versus Oral Medication in the Treatment of Neck Pain: Study Protocol for a Systematic Review and Meta-Analysis
Source: JMIR Res Protoc. 2026 May 18;15:e86168. doi: 10.2196/86168 (PMC13183271; doi:10.2196/86168)

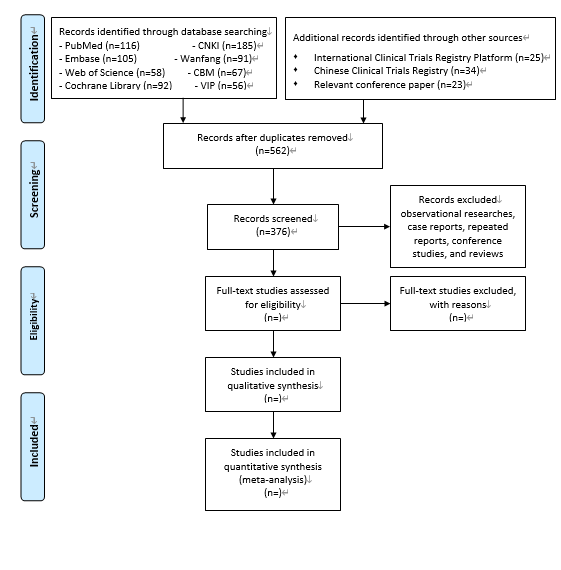

Supplement: Multimedia Appendix 1 [file resprot-v15-e86168-s001.png]
